# Supplementary material for: Behavioral evidence for the hierarchical execution of sequential movements
Source: Commun Psychol. 2026 Mar 11;4:52. doi: 10.1038/s44271-026-00436-5 (PMC13009195; doi:10.1038/s44271-026-00436-5)
Supplement: Supplementary file 2 — Supplementary materials [file 44271_2026_436_MOESM2_ESM.pdf]

Supplementary materials for "Behavioral evidence for the hierarchical execution of sequential movements"

*Cuevas Rivera, Darío[1,2] and Kiebel, Stefan J.[1,2]

[1]Chair of Cognitive Computational Neuroscience, Faculty of Psychology, Technische Universität Dresden, 01062 Dresden, Germany.
[2]Centre for Tactile Internet with Human-in-the-Loop (CeTI)
*Corresponding author: Dario Cuevas Rivera, dario.cuevas_rivera@tu-dresden.de

Participant data

Data for all participants, shown in the same format as in the main text. Note that the single-target data for participant 402 is missing due to technical reasons, as discussed in the main text.

Each of the figures follows the following format (see the caption of Figure 2 of the main text): Movement trajectories for two representative participants. Participant A's trajectories are shown in the first two columns, and those of participant B in the last two. Participants A and B are labeled on top of each figure (e.g. 401 and 402 for the first one). Each panel shows two different movements, and the corresponding speed profiles at the bottom. Each individual line represents one trial, using the MoveID-based color coding. Note that for the two single-target movements, the speed profiles overlap significantly, with the trajectories towards target 1 (blue) plotted behind the trajectories for target 2 (red). The black lines connect the centers of all relevant pairs of circles, for reference.

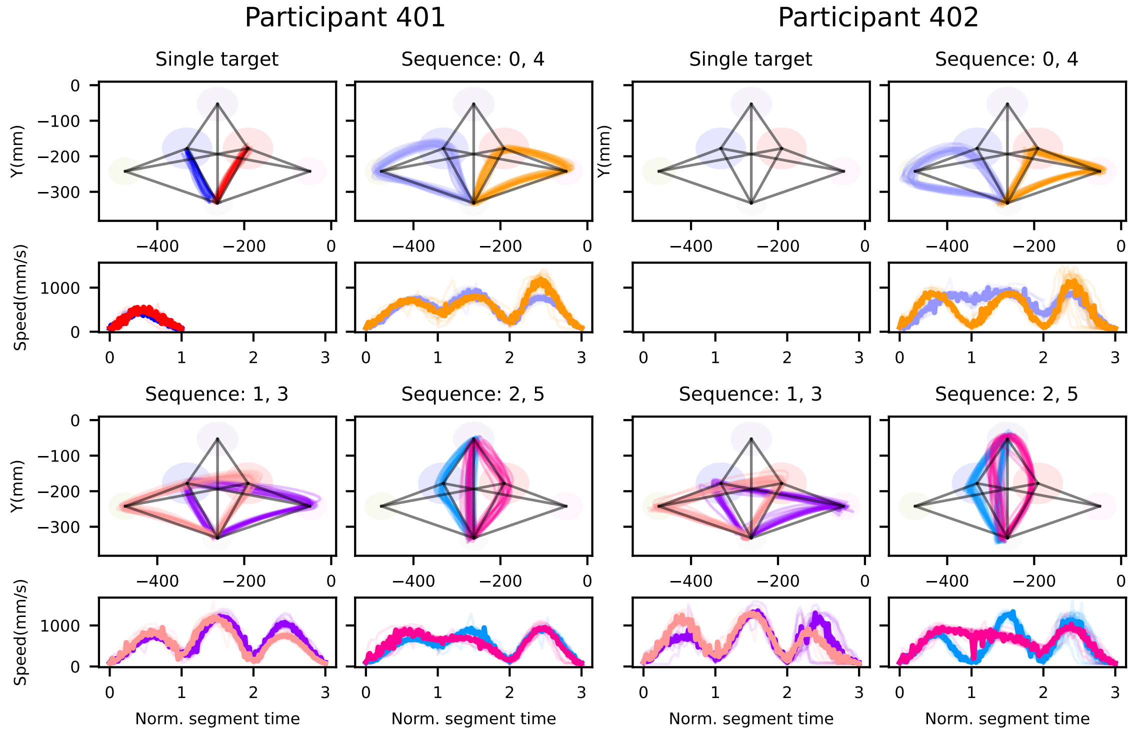
Participant 401
Participant 402
Single target
Sequence: 0, 4
Sequence: 1, 3
Sequence: 2, 5
Y(mm)
Speed(mm/s)
Norm. segment time

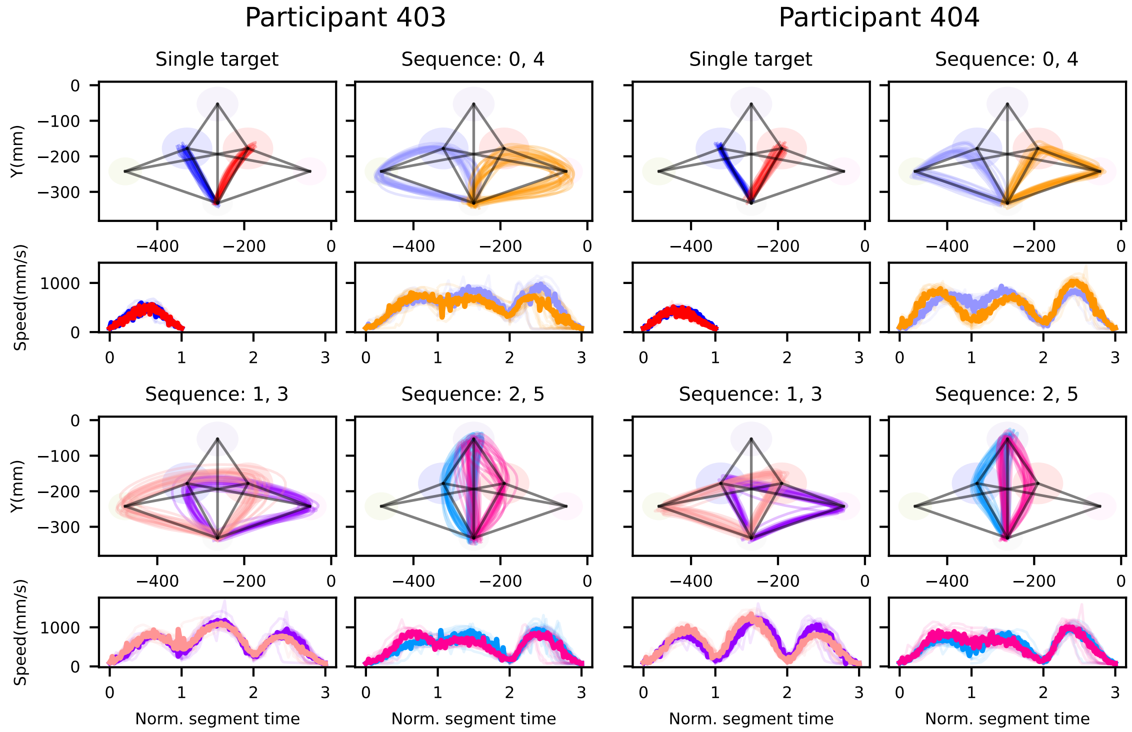
Participant 403
Participant 404
Single target
Sequence: 0, 4
Sequence: 1, 3
Sequence: 2, 5
Y(mm)
Speed(mm/s)
Norm. segment time

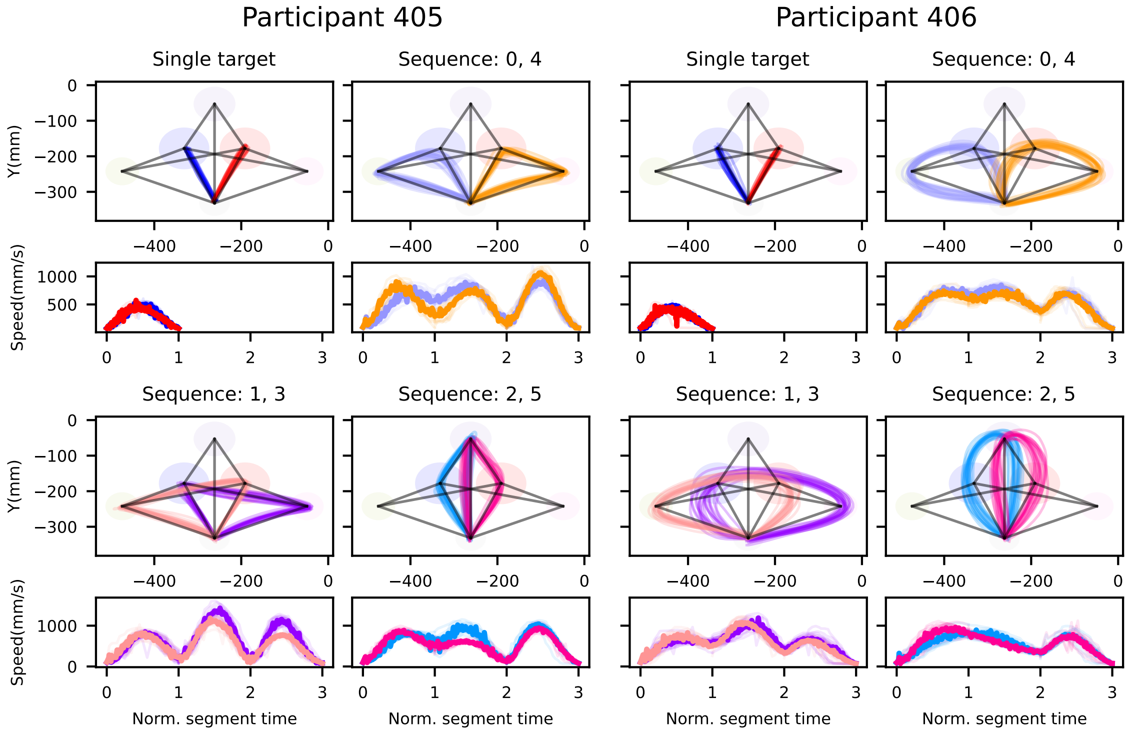
Participant 405
Participant 406
Single target
Sequence: 0, 4
Sequence: 1, 3
Sequence: 2, 5
Y(mm)
Speed(mm/s)
Norm. segment time

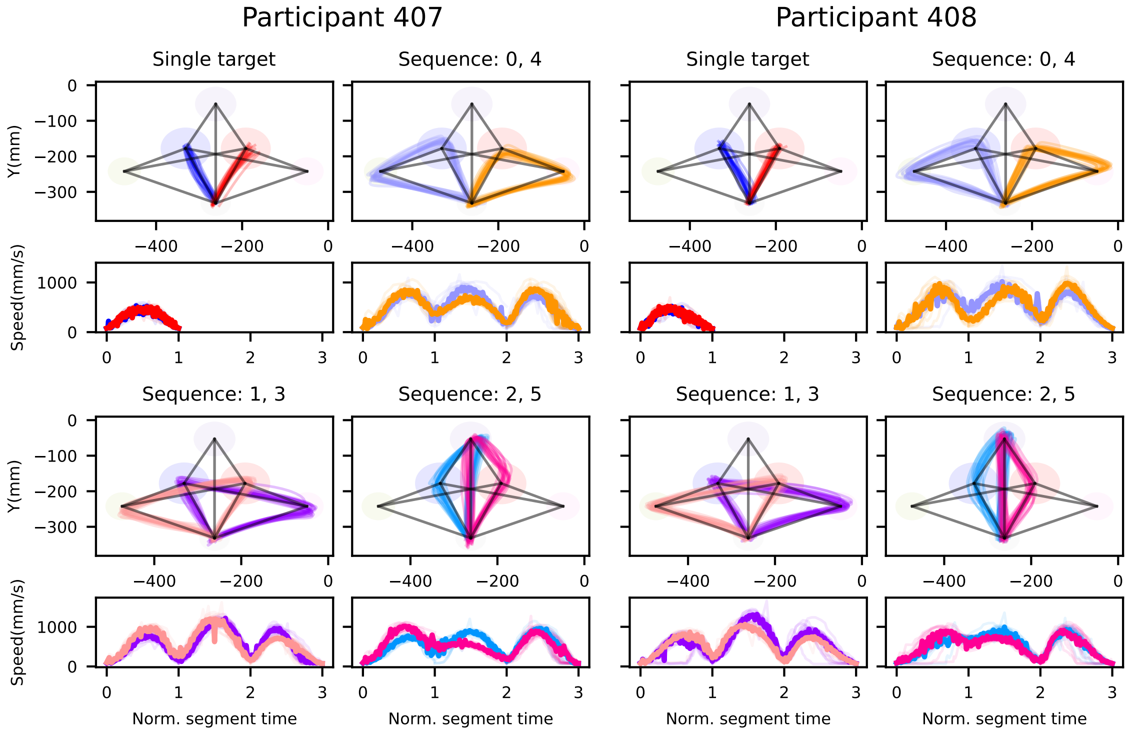
Participant 407
Participant 408
Single target
Sequence: 0, 4
Sequence: 1, 3
Sequence: 2, 5
Y(mm)
Speed(mm/s)
Norm. segment time

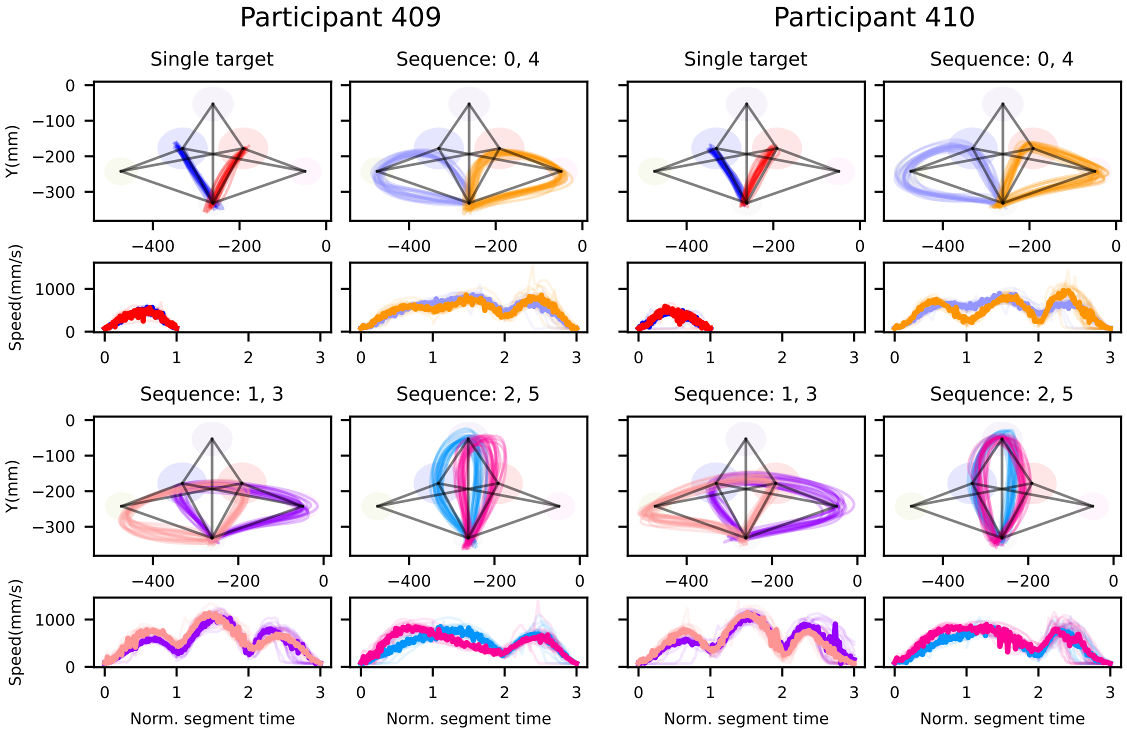
Participant 409
Participant 410
Single target
Sequence: 0, 4
Sequence: 1, 3
Sequence: 2, 5
Y(mm)
Speed(mm/s)
Norm. segment time

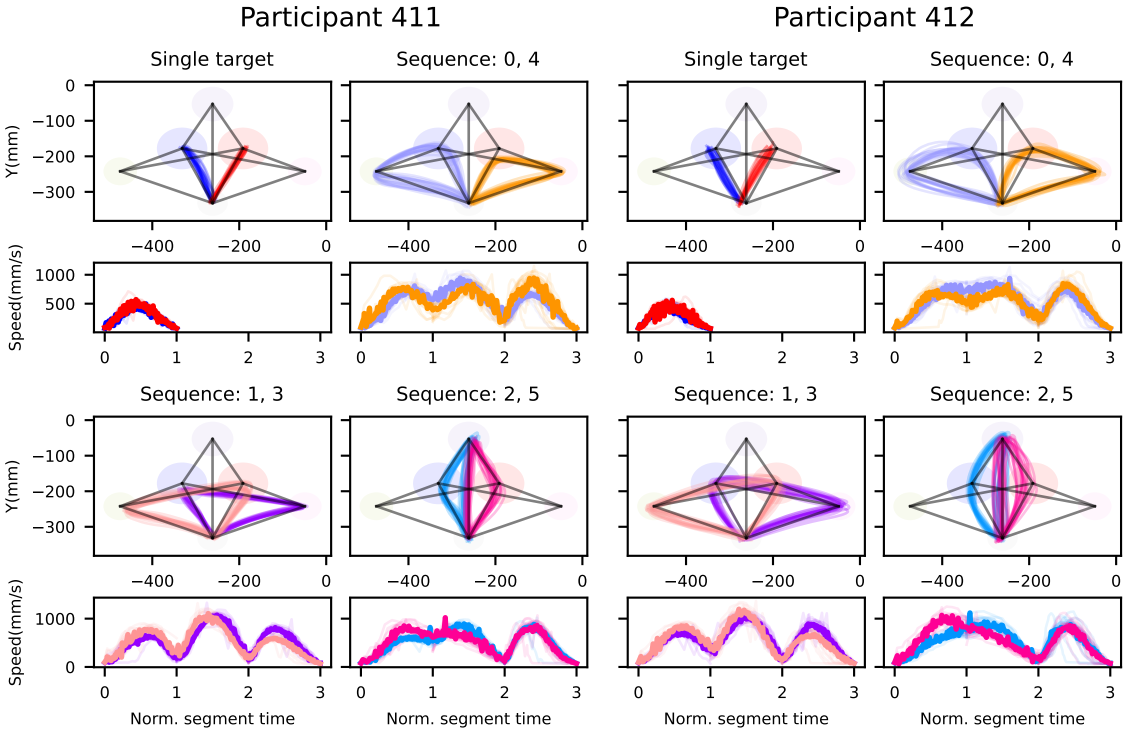
Participant 411
Participant 412
Single target
Sequence: 0, 4
Sequence: 1, 3
Sequence: 2, 5
Y(mm)
Speed(mm/s)
Norm. segment time

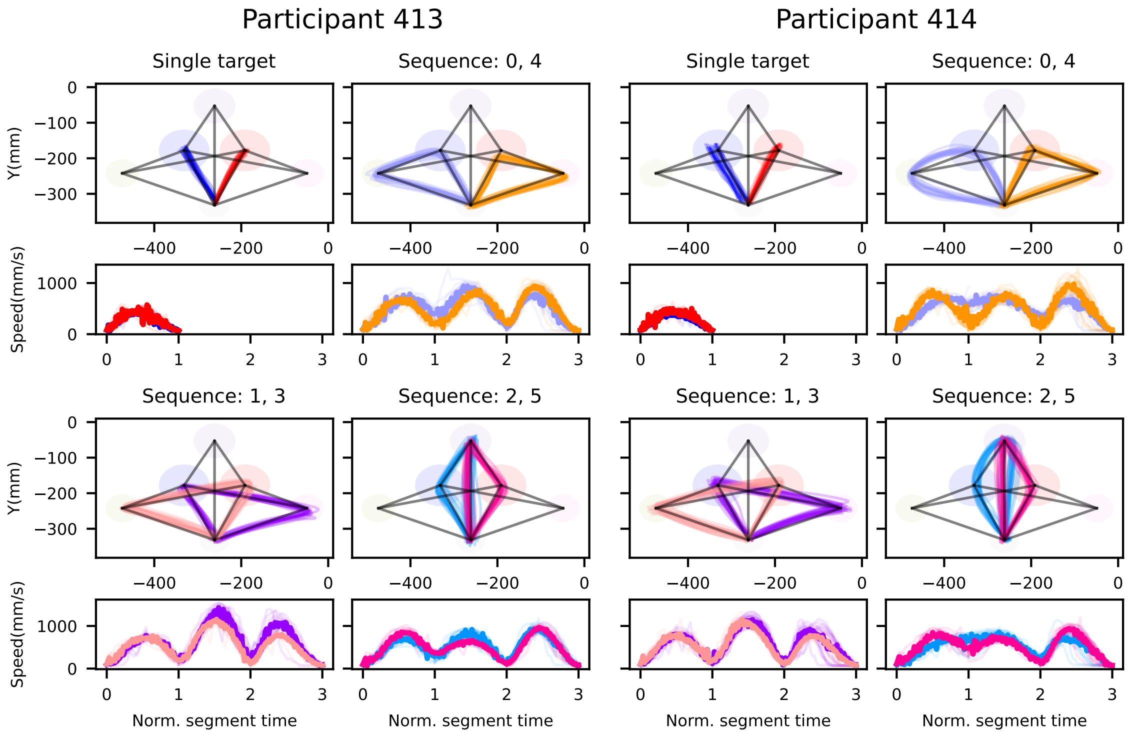
Participant 413
Participant 414
Single target
Sequence: 0, 4
Sequence: 1, 3
Sequence: 2, 5
Y(mm)
Speed(mm/s)
Norm. segment time

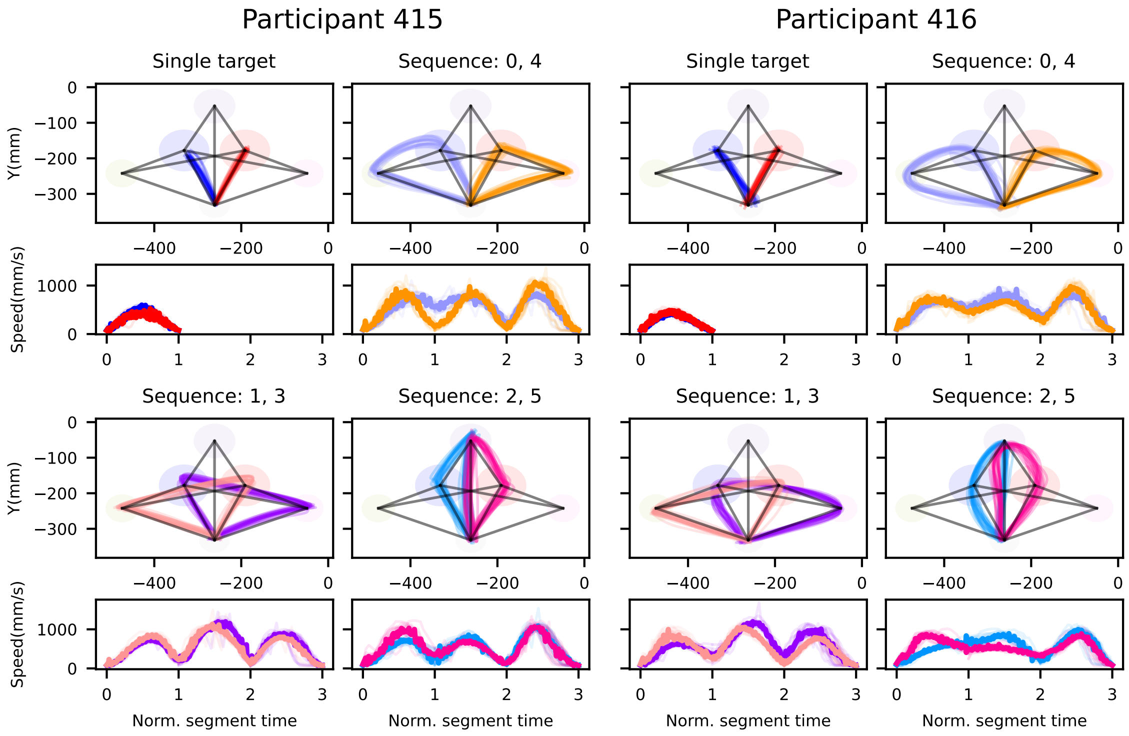
Participant 415
Participant 416
Single target
Sequence: 0, 4
Sequence: 1, 3
Sequence: 2, 5
Y(mm)
Speed(mm/s)
Norm. segment time

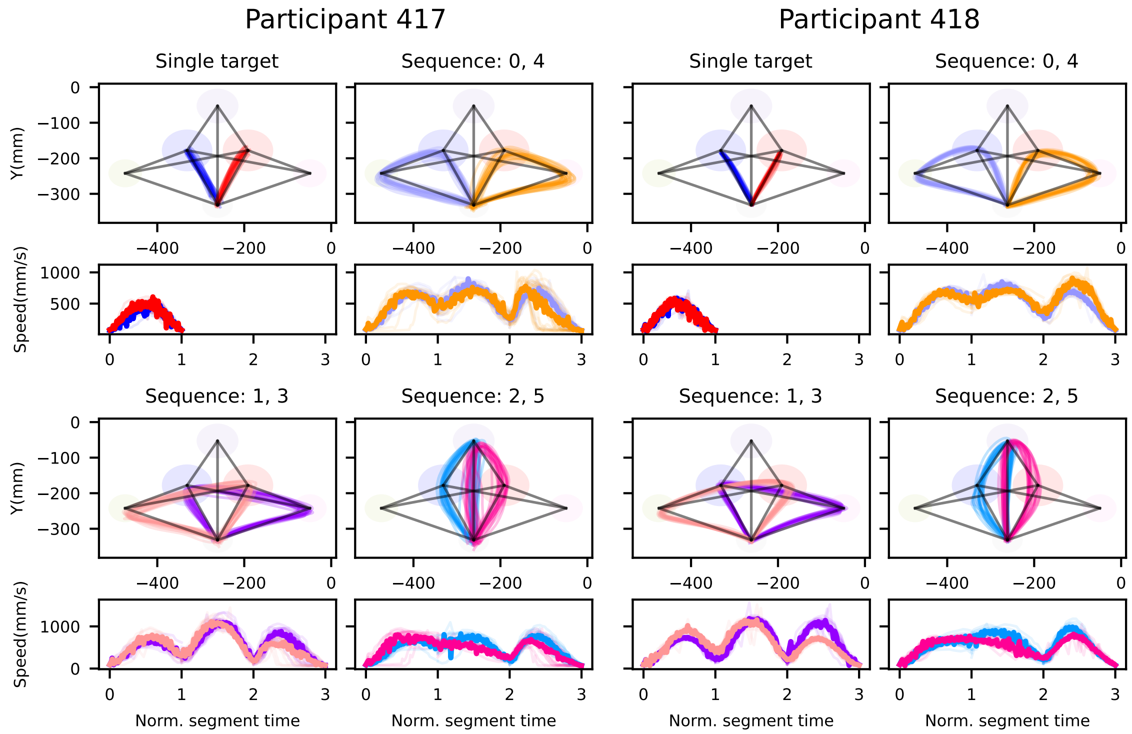
Participant 417
Participant 418
Single target
Sequence: 0, 4
Sequence: 1, 3
Sequence: 2, 5
Y(mm)
Speed(mm/s)
Norm. segment time

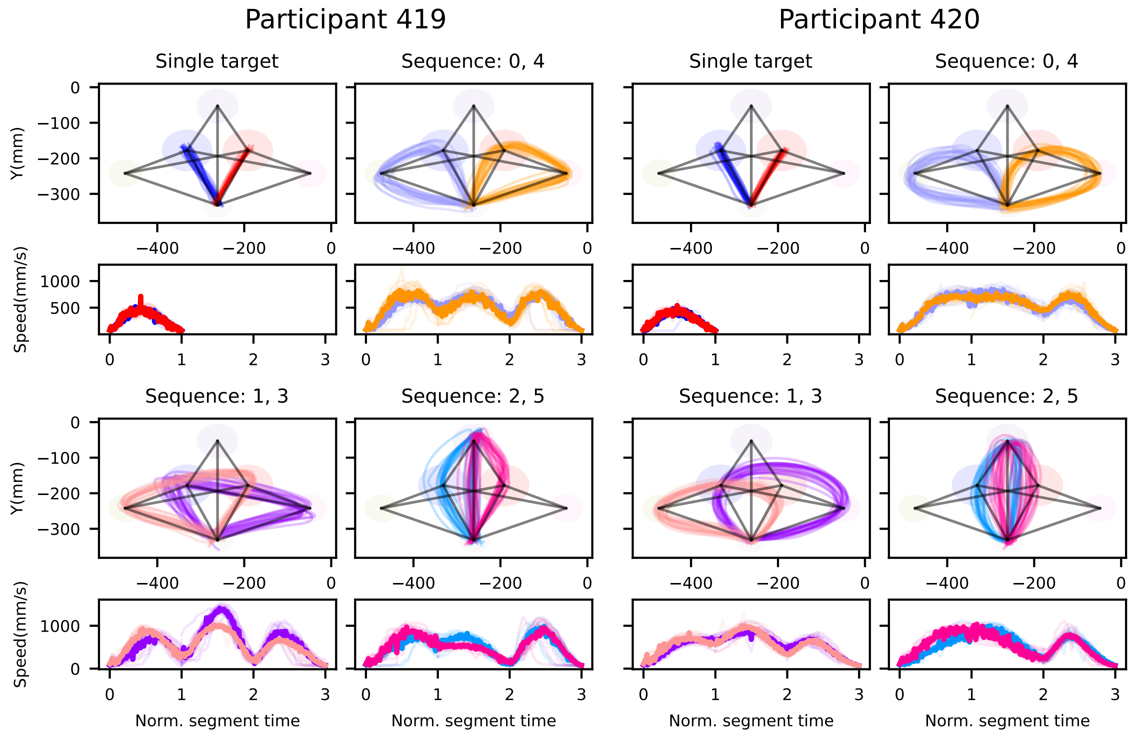
Participant 419
Participant 420
Single target
Sequence: 0, 4
Sequence: 1, 3
Sequence: 2, 5
Y(mm)
Speed(mm/s)
Norm. segment time

vpSOC model

In vpSOC, the multiple targets in a sequence are encoded in the matrices Q_t, each one an (N, N) matrix that encodes, for each time $0 < t \leq T$, a desired state (x, y) (i.e., $N = 2$) to visit at time t. In addition, this matrix incorporates assumptions about the noise in the motor system, both additive and multiplicative. For more details, see the full model specification in the Methods section. Any number of targets can be encoded in a single Q_t (limited only by the number of timesteps t); in this section, we show simulations performed with vpSOC with one, two and three targets. For what follows, we use the name vpSOC(N) to refer to a vpSOC with N targets.

Importantly, similar to the h_{scaling} parameter by [10], we used a set of parameters h_i which determine, for each target i in a sequence, the relative importance of visiting this target exactly at time t_i: a large value for h_i creates trajectories that attempt to cross the exact point (e.g. the center of the circle) exactly at time t_i, greatly reducing variability at the expense of additional exerted control.

As the target-specific precision h_i is a key parameter to relax the requirement to exactly reach an intermediate target, we will use h_i to model coarticulation effects. Another model parameter rarely discussed in previous studies is r, a scalar that establishes the cost of control (see Methods). The higher r is, the more the agent will try to minimize exerted control, i.e. avoid higher accelerations. We will show in the remainder of this section how most of the different planning strategies discernible from the observed trajectories can be modeled with vpSOC by changing the values of h_i and r, while keeping all other parameters fixed. For the values of these parameters, see Methods.

vpSOC simulations and parameter values

As was shown by [10], the SOC model can reproduce simple reaching movements. We compared simulations from vpSOC(1) with our experimental data to determine the value of the additive noise parameter of the plant for the rest of the simulations. We found that these noise parameters led to trial-by-trial variability around targets (i.e. transitional crosses) that was too low compared to the observed sequential movements of our participants. We show this in sample simulations in Supp. Figure 1A (compare the first column with the second and third columns). This mismatch between simulations and experimental data is a property of the vpSOC model when limited to a linear plant model, as implemented by [10], and is due to the extra control exerted by the model in order to reach the intermediary targets. Relaxing the demands for precision (i.e. the h_i-scaling parameter in the model) allows for more variability in sequential trajectories, reducing the mismatch in variability between the model's trajectories and those from participants. However, as we show below, these precision parameters are key for determining the sign and size of the observed coarticulation in the simulated trajectories. Therefore, the h_i parameters cannot be used freely to adjust variability in trajectories without creating a mismatch between the coarticulation in simulated and observed trajectories.

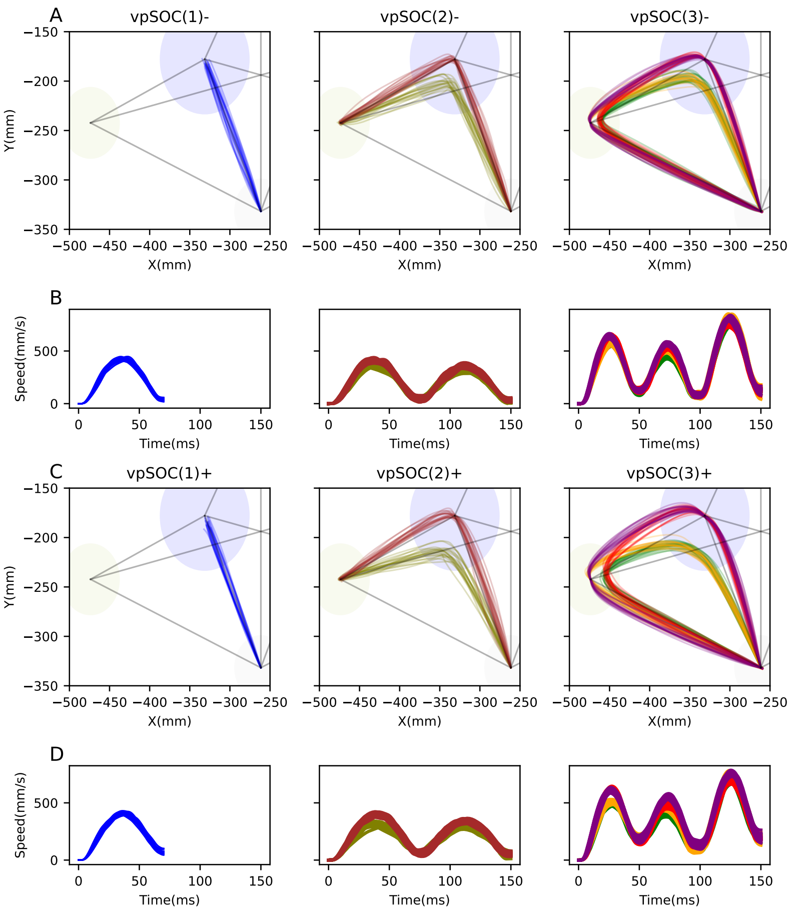

Supp. Figure 1. Simulations with the vpSOC model (A) Single-target (left) movements simulated with vpSOC(1)-, 2-target movements simulated with vpSOC(2)- (middle) and 3-target movements with vpSOC(3)- (right), where the minus signs in the names refers to the low cost of control parameter value r and four combinations of target-specific precision values (h_{blue}, h_{olive}): brown for (high, high), red for (high, low), olive for (low, high) and green for (low, low). (B) Speed profiles for the movements in A. (C) Same as A, but with a high cost of control parameter value r. (D) Speed profiles for C.

An important aspect of the simulations in Supp. Figure 1 is the difference between vpSOC(2) and vpSOC(3) trajectories. When comparing the columns in Supp. Figure 1, it can be seen that the trajectories produced by these three models display different geometries even in the segments they have in common, depending on the parametrization (especially the h parameters). These differences, which we discuss below, will be crucial in establishing HiSeq in the following section, as it points towards the need to link vpSOC(1) and vpSOC(2) as building blocks for longer sequences.

As can be seen in Supp. Figure 1, the vpSOC(1) model will only produce rather straight lines, greatly differing from vpSOC(2) and vpSOC(3). Furthermore, an important difference between vpSOC(2) and vpSOC(3) can be observed around the second target: vpSOC(2) produces trajectories that move in a straight line towards the second target, regardless of parametrization (the h_i parameters were selected to roughly match the hC^1 and tC^1 between vpSOC(2) and vpSOC(3) for all simulations). This is because, for these 2-target sequences, the second target is the last one. The same can be observed for vpSOC(3) towards the third target. In contrast, vpSOC(3) produces trajectories that show anticipatory coarticulation before the second target, as the switch to the third target is being prepared. This can be most easily seen in Supp. Figure 1C, where coarticulations (halfway and transitional) are high overall. As we will show in the following sections, many participant trajectories follow the geometry observed in vpSOC(2) for the first two targets, then transitioning to a last segment back to the initial position. The resulting multi-target movement cannot be reproduced by vpSOC(3) alone.

The effects of the r parameter on the vpSOC model can be seen in Supp. Figure 1A-B, where a low value of r (i.e. a low cost of control) leads to sharp turns around targets. In contrast, Supp. Figure 1C-D shows the same simulations with a high cost of control r, with rounded transitions from the first target to the second. This difference follows intuition, as sharp turns, while allowing higher precision in reaching the first target, come at a high expenditure of control. We will show, in the following sections, that many participant trajectories show a mix between the simulated trajectories with high and low r values, contrary to the interpretation of the r parameter, which suggest it should be constant throughout one movement.

A hierarchical model for sequence planning: HiSeq

In this section, we propose a hierarchical model for planning and execution of sequential movements that addresses the issues with vpSOC discussed in the previous section. This model was motivated by two lines of thought: (1) the high initial cost of planning long movements with vpSOC, and (2) evidence from neuroimaging studies in humans and non-human animals. In what follows, we show how this model expands on the explanatory power of vpSOC by showing how it fits facets of our participants' data that vpSOC cannot.

Hierarchical sequential (HiSeq) model

We propose a three-level hierarchical model for sequential movement planning, HiSeq. The top level represents decision making (i.e. which known sequence to execute); the middle level represents sequential planning (i.e. the order of the sequence); The bottom level represents movements themselves (i.e. muscle activation to reach a target). At the lowest level of the hierarchy, we use vpSOC models for both single-target and simple 2-target movements. While vpSOC(3) can also be used as building blocks, our HiSeq simulations here do not include this possibility, as it would simply produce the same results as vpSOC(3) alone. The second level dynamically concatenates these vpSOC building blocks into sequences of movements. Crucially, this second level can store and execute different sequences of simple movements. The highest level selects one of these sequences by setting the connectivity between the different elements of the second level, following [1].

More specifically, the middle level is modeled by a high-dimensional dynamical system, where each one of the possible elements (movements) is encoded as an saddle equilibrium point given by the generalized Lotka-Volterra equations. These and similar equations have been used before to model sequential activity in the brain [5, 2, 7, 8, 3], for inferring behavior [6] and for movement in robotics [9, 4] by taking advantage of the reproducibility and resistance to noise of the trajectories they produce, a phenomenon called the stable heteroclinic channel (SHC); for details, see [1]. In particular, the parametrization of these equations presented by [1] guarantees that a given sequence of equilibrium points is visited regardless of noise in the dynamics. In our implementation, once an element in a sequence (i.e. an equilibrium point) has been reached, the transition to the next is triggered by a component that monitors when a target (i.e. a circle) has been reached in kinematic space. This monitor then sends a target- and sequence-agnostic pulse to the SHC, triggering the transition to the next equilibrium point.

Each of the equilibrium point at the second level links to a vpSOC at the lowest level. In our task, each one of these simple models represents the basic movement to one or more circles in our task. The currently active vpSOC and its corresponding equilibrium point is activated at the second level in a sequential and temporally-overlapping fashion. The final motor commands are assumed to be a weighted average over all the motor commands of the active vpSOCs. This online mixing of vpSOCs is what creates transitional coarticulation independent of the dynamics of the individual vpSOC elements. For a full description of the model, see Methods.

Features of HiSeq

The HiSeq model expands on the explanatory power of vpSOC alone, as it uses vpSOC models as building blocks. Importantly HiSeq relies on these pre-learned building blocks to create sequential movements. The importance of this lies in the cost of planning: HiSeq does not need to re-compute the controller each time a new sequence is required. In contrast, with vpSOC the previously-calculated controllers cannot be used for a new sequence, even if that

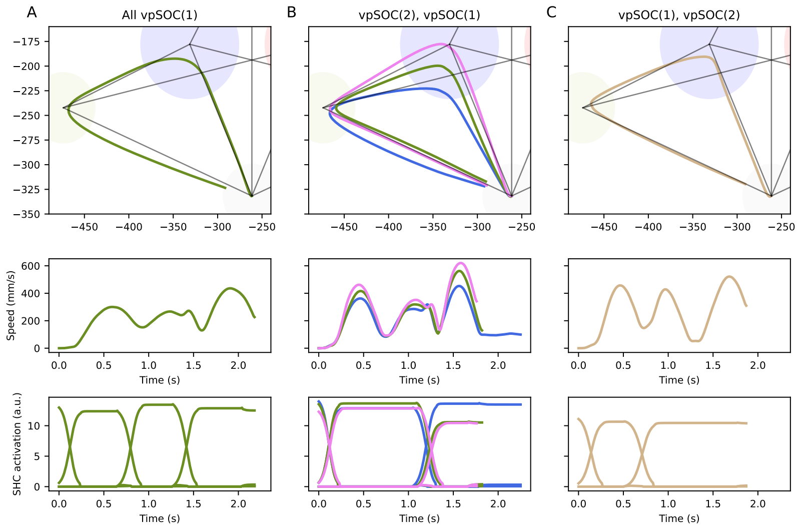

Supp. Figure 2. Hiseq simulations. (A) Simulations with a HiSeq consisting of single-target SOC models for each segment. The top panel shows trajectories of MoveID 0, simulated without noise. The second panel below shows the movement speed, and the bottom panel shows the evolution of the SHC. (B) Simulations with a HiSeq consisting of a vpSOC(2) and a vpSOC(1) to return to the starting position, in the sdame format as A (D) A HiSeq consisting of a vpSOC(1) to reach the first target, and a vpSOC(2) to reach the second target and return to starting position, in the same format as A.

sequence includes some of the same movements as before. Additionally, HiSeq comes with a dynamic flexibility that vpSOC lacks, as targets can be added at the end of a sequence dynamically, simply by adding extra connections to the last element in the SHC layer. With this, and again in contrast with vpSOC, no new controllers need to be computed.

In Supp. Figure 2A we show a diagram of the interaction between the two lowest levels of HiSeq: in the simplest case, a two-target sequence can be modeled in HiSeq as the concatenation of two single-target vpSOC(1) movements. In isolation, i.e. not embedded in HiSeq, single-target vpSOC models create straight-line movements. When concatenated by embedding into HiSeq, the same two single-target vpSOCs generate a trajectory with high transitional coarticulation due to the overlap of the two SHC elements, as shown in Supp. Figure 2A. This architecture grants the model flexibility in mixing different types and signs of coarticulation within one sequence. In contrast to vpSOC, HiSeq is capable of disentangling halfway variability from transitional variability. This occurs through two interacting mechanisms: (1) the internal noise of the sequential dynamics at the 2nd level (SHC, in our implementation), and (2) the exact timing of each element of the SHC.

As illustrative examples, we simulated a sequence (MoveID 0; see Fig. 1C) with HiSeq, using different building blocks and parametrization to show the flexibility of the model. In Supp. Figure 2A, we show a HiSeq model comprising vpSOC(1) building blocks, where coarticulation (both halfway and transitional) is created by the SHC component. In Supp. Figure 2B, we show a HiSeq in which the first two targets are reached with a vpSOC(2), and the third is dynamically-linked vpSOC(1). In this case, hC^1 is determined by both the SHC

and vpSOC(2) components, while tC^1 is directly determined by vpSOC(2). We simulated different parametrizations to show the different mixtures of coarticulation (different colors of trajectories in Supp. Figure 2C) we found in the participant data, where higher tC^1 and positive hC^1 were obtained with lower h_{blue} Finally, in Supp. Figure 2C we show trajectories obtained with a HiSeq consisting of vpSOC(1)+vpSOC(2), where the vpSOC(2) for the second and third targets causes negative hC^2 and hC^3, combined with the near-zero positive hC^1.

One important consequence of the hierarchical generation of sequences is that the cost of control is not necessarily minimized throughout the entire trajectory. With vpSOC(N), the trajectories are planned to minimize the cost function (i.e. cost of control) throughout all N targets, leading to the dichotomy observed in Supp. Figure 1, in which trajectories are either sharp turns (with low r) around all targets, or rounded trajectories. HiSeq, in contrast, can produce e.g. very rounded (e.g. low cost of control) trajectories around the first target, and sharp turns around the second, as in Supp. Figure 2B (in particular the blue trajectory), similar to those shown in Supp. Figure 3(top-right). It is these sharp turns that increase the cost of control of a sequential movement in HiSeq. In what follows, we will show that there is evidence of such varying planning costs in the participants' observed trajectories.

Modeling coarticulation with vpSOC and HiSeq

We first qualitatively compare vpSOC and HiSeq with participant data by focusing on different types and signs of coarticulation. We begin with the sequence blue-green-start (MoveID 0). For the first segment of this sequence, the parameters h_{blue} and h_{green} control the coarticulation in the first segment (from the starting point to the blue circle). The involvement of h_{green} (associated with the second green target) in the coarticulation during the first segment is a property of vpSOC, whereby trajectories during the first segment are modified in anticipation of the trajectories towards the second segment.

In Supp. Figure 3, we show a grid of data and simulations, where each row represents one MoveID (0, 2 and 3, respectively). Each column represents one specific model, starting with vpSOC(3) with high energy cost r (vpSOC(3)+), vpSOC(3)- with low r, and a HiSeq consisting of one vpSOC(2) for the first two targets, and one vpSOC(1) for the last one. Representative participant data was selected for each row to showcase characteristics that only one of the three shown models can simulate. Different participant-MoveID combinations were chosen for each panel, and the full list can be seen in the Methods section. vpSOC(3)+ produces trajectories with high negative halfway coarticulation in all segments, which pass through the centers of all targets with high precision. In contrast, vpSOC(3)- reproduces trajectories with sharp turns and near-zero halfway coarticulation. HiSeq reproduces trajectories with negative halfway coarticulation in the first segment (hC^1), with a round transition from first to second targets, and high precision on the first target (reaching its center).

The columns in Supp. Figure 3 are arranged such that the first column contains data

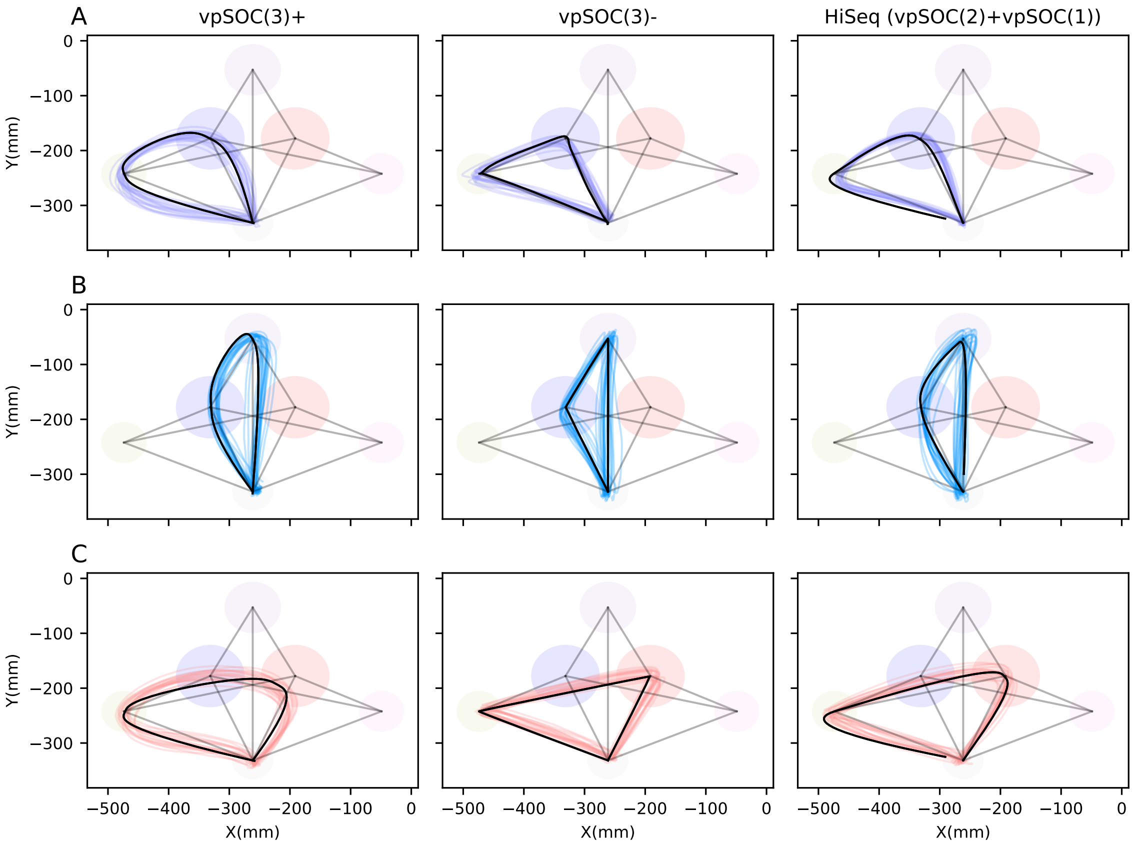

Supp. Figure 3. Participant data and simulations. (A) Simulations with three models (vpSOC(3)+, vpSOC(3)- and HiSeq; black lines) alongside all repetitions from one participant in MoveID-based color coding, for MoveID 0. The data comes from different participants. (B) Same as A, for MoveID 2. (C) Same as A, for MoveID 3.

that is best described by vpSOC(3), having, for example, all-negative halfway coarticulation in all segments. Such a property cannot be obtained from simulations of HiSeq (except for trivially using a single-element SHC tied to a vpSOC(3) building block). The second column contains trajectories that can be simulated both with vpSOC(3) (with low r) or with HiSeq (with three vpSOC(3) elements). The third column contains trajectories that cannot be simulated with vpSOC(3) due to the mix of hC signs across segments, as well as the sharp turns around the second target.

The trajectories in Supp. Figure 3 were chosen for two reasons: (1) we believe they represent the variability in our participant data, and (2) because they highlight the capacity of the two models to reproduce the observed geometries.

	p left	p right	d left	d right	CI left	CI right	M left	M right
401	0.0000	0.0000	1.3625	1.6725	[5.73, 11.72]	[5.61, 9.98]	8.7226	7.7952
403	0.0001	0.0000	1.1228	1.5904	[4.13, 10.04]	[7.07, 12.96]	7.0829	10.0160
404	0.4138	0.0017	0.1868	0.8162	[-2.34, 1.00]	[2.43, 8.97]	-0.6677	5.7018
405	0.0001	0.0001	1.1186	1.0768	[-5.76, -2.36]	[2.64, 6.69]	-4.0602	4.6655
406	0.0684	0.0045	0.4319	0.7208	[-0.20, 5.07]	[0.90, 4.23]	2.4322	2.5653
407	0.0785	0.1472	0.4158	0.3379	[-0.37, 6.33]	[-0.84, 5.23]	2.9788	2.1932
408	0.0000	0.0000	1.8760	1.3374	[-15.57, -9.35]	[3.60, 7.48]	-12.4608	5.5438
409	0.0096	0.0010	0.6443	0.8699	[0.83, 5.22]	[2.81, 9.34]	3.0264	6.0749
410	0.2398	0.0000	0.2714	1.7068	[-1.00, 3.77]	[9.61, 16.87]	1.3831	13.2411
411	0.0000	0.3753	2.2527	0.2030	[-14.19, -9.31]	[-0.91, 2.31]	-11.7474	0.6989
412	0.0000	0.0000	2.6431	2.6523	[13.08, 18.71]	[13.76, 19.65]	15.8943	16.7053
413	0.0083	0.0000	0.6589	1.6791	[-4.82, -0.82]	[3.94, 6.99]	-2.8176	5.4625
414	0.0025	0.0000	0.7793	1.4487	[2.33, 9.34]	[4.24, 8.29]	5.8331	6.2634
415	0.0000	0.2508	1.6333	0.2649	[-11.74, -6.51]	[-0.75, 2.70]	-9.1225	0.9745
416	0.0000	0.0000	2.1938	1.3724	[-10.52, -6.82]	[4.36, 8.87]	-8.6731	6.6132
417	0.0002	0.0000	1.0249	1.8574	[-7.34, -2.74]	[7.09, 11.87]	-5.0404	9.4835
418	0.0000	0.6028	1.7196	0.1183	[-6.80, -3.89]	[-1.25, 2.10]	-5.3485	0.4238
419	0.0186	0.0000	0.5752	1.4489	[-6.13, -0.63]	[3.65, 7.13]	-3.3822	5.3877
420	0.0521	0.1085	0.4634	0.3765	[-0.03, 6.25]	[-0.40, 3.67]	3.1108	1.6351

Supp. Table 1
Halfway coarticulation significantly different from zero. For each participant, p-values and Cohen's d for the t-tests showing whether the trajectories starting with the blue circle (left), or the red circle (right), show coarticulation that is significantly different from zero. Values lower than 0.01 reject the null hypothesis that trajectories show no halfway coarticulation. Shown are the p-values (p left/right), Cohen's d (d left/right), 95% confidence intervals (CI left/right) and means (M left/right).

Statistical tests

In this section, we present all statistical tests performed on data. In the main article, only a summarized version of these tables is shown, and each table is referenced individually.

Supp. Table 2
Halfway coarticulation significantly different from zero, separated by participant and sequence. Shown are p-values, Cohen's d, the mean M and the 95% confidence interval.

Part.	Seq.	p	d	CI	M
401	0	0.0021	0.5206	[1.70, 7.10]	4.3976
402	0	0.0056	0.6985	[1.62, 8.20]	4.9096
403	0	0.0018	0.5313	[-17.47, -4.34]	-10.9060
404	0	0.0226	0.3755	[0.41, 5.08]	2.7462
405	0	0.0272	0.3628	[-4.20, -0.26]	-2.2320
406	0	0.0001	0.6692	[-13.81, -4.88]	-9.3455

Continued on next page

Supp. Table 2

Halfway coarticulation significantly different from zero, separated by participant and sequence. Shown are p-values, Cohen's d, the mean M and the 95% confidence interval.

Part.	Seq.	p	d	CI	M
407	0	0.2462	0.1862	[-3.78, 1.00]	-1.3918
408	0	0.0000	0.9806	[-10.82, -5.50]	-8.1576
409	0	0.0653	0.2999	[-4.51, 0.15]	-2.1834
410	0	0.5031	0.1069	[-3.36, 1.67]	-0.8403
411	0	0.0000	2.1405	[-14.06, -10.40]	-12.2294
412	0	0.0620	0.3039	[-0.21, 8.29]	4.0401
413	0	0.0001	0.6744	[-4.38, -1.56]	-2.9707
414	0	0.0439	0.3293	[-7.35, -0.11]	-3.7265
415	0	0.0000	1.9998	[-14.72, -10.66]	-12.6935
416	0	0.0000	1.2434	[-10.39, -6.14]	-8.2615
417	0	0.0005	0.5960	[-5.01, -1.51]	-3.2609
418	0	0.0000	1.7155	[-11.91, -8.16]	-10.0348
419	0	0.2445	0.1868	[-3.74, 0.98]	-1.3777
420	0	0.2213	0.1965	[-4.75, 1.13]	-1.8083
401	1	0.4233	0.1279	[-4.74, 2.03]	-1.3554
402	1	0.0000	3.3392	[-21.05, -15.87]	-18.4623
403	1	0.1379	0.2395	[-9.23, 1.33]	-3.9524
404	1	0.0000	1.0343	[4.64, 8.79]	6.7127
405	1	0.6650	0.0690	[-1.52, 2.35]	0.4177
406	1	0.0000	0.8280	[-39.12, -17.32]	-28.2199
407	1	0.2026	0.2049	[-4.00, 0.88]	-1.5629
408	1	0.0503	0.3194	[-0.00, 4.03]	2.0160
409	1	0.0105	0.4249	[-10.40, -1.47]	-5.9360
410	1	0.2523	0.1837	[-9.36, 2.53]	-3.4146
411	1	0.0488	0.3216	[-2.95, -0.01]	-1.4798
412	1	0.4099	0.1317	[-9.37, 3.90]	-2.7335
413	1	0.0000	0.8446	[2.24, 4.96]	3.5989
414	1	0.0547	0.3132	[-0.05, 4.54]	2.2461
415	1	0.9795	0.0041	[-1.37, 1.33]	-0.0173
416	1	0.0055	0.4652	[-11.50, -2.13]	-6.8150
417	1	0.7783	0.0448	[-4.13, 3.11]	-0.5077
418	1	0.0577	0.3092	[-2.33, 0.04]	-1.1470
419	1	0.0002	0.6531	[-19.37, -6.63]	-13.0008
420	1	0.0000	0.8408	[-20.84, -9.35]	-15.0950
401	2	0.0000	2.0292	[-11.09, -6.93]	-9.0131
402	2	0.0000	3.6088	[-21.02, -16.19]	-18.6082
403	2	0.0000	2.2075	[-19.00, -12.35]	-15.6790
404	2	0.0051	0.7083	[-8.89, -1.82]	-5.3511
405	2	0.0000	1.3045	[-9.71, -4.58]	-7.1435

Continued on next page

Supp. Table 2

Halfway coarticulation significantly different from zero, separated by participant and sequence. Shown are p-values, Cohen's d, the mean M and the 95% confidence interval.

Part.	Seq.	p	d	CI	M
406	2	0.0000	2.4708	[-29.35, -20.00]	-24.6755
407	2	0.0000	1.8317	[-12.80, -7.59]	-10.1959
408	2	0.0012	0.8482	[-8.33, -2.41]	-5.3707
409	2	0.0000	1.9934	[-18.41, -11.41]	-14.9078
410	2	0.0000	1.7465	[-15.78, -9.11]	-12.4460
411	2	0.6324	0.1087	[-3.23, 2.01]	-0.6080
412	2	0.0000	7.3946	[-26.37, -23.23]	-24.8020
413	2	0.4142	0.1867	[-5.46, 2.35]	-1.5563
414	2	0.0111	0.6294	[-7.06, -1.04]	-4.0489
415	2	0.7309	0.0780	[-3.34, 2.39]	-0.4776
416	2	0.0000	3.2023	[-26.90, -20.04]	-23.4663
417	2	0.0000	2.9771	[-18.40, -13.40]	-15.8993
418	2	0.0000	2.2609	[-19.29, -12.67]	-15.9785
419	2	0.0000	1.2875	[-20.72, -9.67]	-15.1973
420	2	0.0000	3.2716	[-29.16, -21.86]	-25.5064
401	3	0.8752	0.0356	[-2.36, 2.75]	0.1941
402	3	0.0000	2.0310	[7.09, 11.34]	9.2147
403	3	0.0000	2.0046	[-32.66, -20.30]	-26.4791
404	3	0.0000	1.8451	[8.75, 14.70]	11.7224
405	3	0.0000	1.8129	[-5.72, -3.37]	-4.5488
406	3	0.0000	4.6490	[-48.09, -39.29]	-43.6896
407	3	0.2671	0.2557	[-3.34, 0.98]	-1.1800
408	3	0.0209	0.5630	[0.41, 4.43]	2.4172
409	3	0.0000	3.2004	[-24.60, -18.32]	-21.4581
410	3	0.0067	0.6801	[1.33, 7.23]	4.2799
411	3	0.0000	2.4974	[-18.36, -12.57]	-15.4648
412	3	0.0000	1.6993	[-12.00, -6.82]	-9.4065
413	3	0.0000	1.3581	[-11.25, -5.48]	-8.3653
414	3	0.2463	0.2675	[-2.83, 0.77]	-1.0295
415	3	0.6086	0.1164	[-1.83, 3.05]	0.6067
416	3	0.0119	0.6223	[-7.19, -1.02]	-4.1055
417	3	0.0000	1.8437	[-12.28, -7.31]	-9.7961
418	3	0.0000	3.4270	[-16.71, -12.69]	-14.6987
419	3	0.0073	0.6719	[0.97, 5.43]	3.1980
420	3	0.0000	2.5518	[-22.11, -15.26]	-18.6833
401	4	0.0000	1.6828	[-14.94, -8.44]	-11.6890
402	4	0.0000	1.4634	[-7.68, -3.96]	-5.8163
403	4	0.0000	1.3588	[-20.16, -9.83]	-14.9920
404	4	0.0022	0.7926	[-9.78, -2.52]	-6.1474

Continued on next page

Supp. Table 2
Halfway coarticulation significantly different from zero, separated by participant and sequence. Shown are p-values, Cohen's d, the mean M and the 95% confidence interval.

Part.	Seq.	p	d	CI	M
405	4	0.0390	0.4957	[0.11, 3.98]	2.0454
406	4	0.0000	3.1354	[-43.87, -32.47]	-38.1698
407	4	0.0000	1.9198	[-11.62, -7.07]	-9.3453
408	4	0.0000	1.9304	[-13.63, -8.31]	-10.9682
409	4	0.0000	2.8870	[-26.04, -18.77]	-22.4049
410	4	0.0000	2.8608	[-21.99, -15.81]	-18.8974
411	4	0.7278	0.0790	[-2.80, 1.99]	-0.4038
412	4	0.0000	4.4379	[-27.64, -22.36]	-24.9999
413	4	0.0009	0.8835	[2.09, 6.80]	4.4469
414	4	0.0000	2.0312	[-10.10, -6.31]	-8.2053
415	4	0.0000	2.0478	[-13.81, -8.67]	-11.2374
416	4	0.0107	0.6333	[-7.39, -1.11]	-4.2472
417	4	0.0007	0.8997	[-12.41, -3.92]	-8.1637
418	4	0.0000	3.2414	[-12.47, -9.32]	-10.8924
419	4	0.0000	2.4604	[-25.43, -17.30]	-21.3691
420	4	0.0000	4.9968	[-35.17, -29.14]	-32.1559
401	5	0.0000	1.3318	[-16.84, -8.08]	-12.4611
402	5	0.7019	0.0869	[-1.71, 2.49]	0.3892
403	5	0.0001	1.1382	[-16.07, -6.70]	-11.3858
404	5	0.0320	0.5175	[0.34, 6.74]	3.5388
405	5	0.0000	1.4783	[-9.43, -4.89]	-7.1625
406	5	0.0000	1.6586	[-20.66, -11.56]	-16.1102
407	5	0.3033	0.2366	[-1.08, 3.30]	1.1086
408	5	0.2801	0.2486	[-5.50, 1.68]	-1.9072
409	5	0.0149	0.5988	[-7.18, -0.88]	-4.0276
410	5	0.1058	0.3797	[-5.62, 0.59]	-2.5191
411	5	0.0000	1.2038	[-10.78, -4.74]	-7.7610
412	5	0.2514	0.2645	[-1.78, 6.39]	2.3090
413	5	0.0000	1.4971	[-7.53, -3.94]	-5.7377
414	5	0.1969	0.2990	[-3.23, 0.71]	-1.2590
415	5	0.0416	0.4887	[-6.11, -0.13]	-3.1230
416	5	0.0000	1.4874	[-12.24, -6.38]	-9.3080
417	5	0.0002	1.0300	[-13.04, -4.89]	-8.9684
418	5	0.0001	1.1538	[-11.67, -4.93]	-8.2990
419	5	0.0549	0.4574	[-0.06, 5.46]	2.7005
420	5	0.1918	0.3026	[-8.05, 1.73]	-3.1602

Supp. Table 3

Significant differences between single-target and sequential movements, separated by participant and sequence. Column 'd' refers to Cohen's d. Means (M sin/seq) and 95% confidence intervals of the difference are shown. NaN entries correspond to participant 402, who was excluded from this analysis because the single-target data was corrupted.

Part.	Seq.	p	d	CI	M sin	M seq
401	0	0.0006	1.1820	[3.96, 13.34]	8.7226	0.0725
401	1	0.0000	3.1756	[15.35, 23.11]	8.7226	-10.5059
401	2	0.0000	3.2191	[14.19, 21.28]	8.7226	-9.0131
401	3	0.0000	1.4985	[4.35, 10.85]	7.7952	0.1941
401	4	0.0000	3.2941	[15.68, 23.29]	7.7952	-11.6890
401	5	0.0000	2.7405	[15.47, 25.04]	7.7952	-12.4611
402	0	NaN	NaN	NaN	NaN	NaN
402	1	NaN	NaN	NaN	NaN	NaN
402	2	NaN	NaN	NaN	NaN	NaN
402	3	NaN	NaN	NaN	NaN	NaN
402	4	NaN	NaN	NaN	NaN	NaN
402	5	NaN	NaN	NaN	NaN	NaN
403	0	0.0000	3.7556	[29.78, 42.18]	7.0829	-28.8949
403	1	0.0000	2.9032	[19.45, 30.56]	7.0829	-17.9208
403	2	0.0000	3.3886	[18.46, 27.06]	7.0829	-15.6790
403	3	0.0000	3.5269	[29.78, 43.21]	10.0160	-26.4791
403	4	0.0000	2.7838	[19.21, 30.81]	10.0160	-14.9920
403	5	0.0000	2.5605	[16.02, 26.79]	10.0160	-11.3858
404	0	0.0028	1.0459	[-11.08, -2.58]	-0.6677	6.1601
404	1	0.0000	1.7079	[-11.56, -5.22]	-0.6677	7.7236
404	2	0.0185	0.7925	[0.85, 8.52]	-0.6677	-5.3511
404	3	0.0070	0.9017	[-10.30, -1.74]	5.7018	11.7224
404	4	0.0000	1.6054	[7.12, 16.58]	5.7018	-6.1474
404	5	0.3287	0.3129	[-2.26, 6.59]	5.7018	3.5388
405	0	0.0622	0.6151	[-7.51, 0.20]	-4.0602	-0.4039
405	1	0.8559	0.0578	[-2.78, 2.32]	-4.0602	-3.8302
405	2	0.0436	0.6637	[0.09, 6.07]	-4.0602	-7.1435
405	3	0.0000	2.6027	[6.93, 11.50]	4.6655	-4.5488
405	4	0.0576	0.6193	[-0.09, 5.33]	4.6655	2.0454
405	5	0.0000	2.5736	[8.88, 14.77]	4.6655	-7.1625
406	0	0.0000	3.2012	[18.82, 28.29]	2.4322	-21.1231
406	1	0.0000	4.3013	[52.07, 70.80]	2.4322	-59.0051
406	2	0.0000	3.3438	[21.87, 32.34]	2.4322	-24.6755
406	3	0.0000	6.5095	[41.62, 50.89]	2.5653	-43.6896
406	4	0.0000	4.5420	[34.86, 46.61]	2.5653	-38.1698
406	5	0.0000	2.5531	[13.90, 23.45]	2.5653	-16.1102
407	0	0.0001	1.4320	[4.82, 12.67]	2.9788	-5.7625

Continued on next page

Supp. Table 3

Significant differences between single-target and sequential movements, separated by participant and sequence. Column 'd' refers to Cohen's d. Means (M sin/seq) and 95% confidence intervals of the difference are shown. NaN entries correspond to participant 402, who was excluded from this analysis because the single-target data was corrupted.

Part.	Seq.	p	d	CI	M sin	M seq
407	1	0.0006	1.1800	[3.80, 12.80]	2.9788	-5.3190
407	2	0.0000	2.0538	[9.06, 17.29]	2.9788	-10.1959
407	3	0.0666	0.5990	[-0.24, 6.99]	2.1932	-1.1800
407	4	0.0000	2.0114	[7.86, 15.22]	2.1932	-9.3453
407	5	0.5485	0.1916	[-2.55, 4.72]	2.1932	1.1086
408	0	0.0005	1.1988	[-13.21, -4.01]	-12.4608	-3.8544
408	1	0.0000	1.7048	[-15.06, -6.84]	-12.4608	-1.5117
408	2	0.0014	1.0927	[-11.24, -2.94]	-12.4608	-5.3707
408	3	0.0245	0.7409	[0.42, 5.83]	5.5438	2.4172
408	4	0.0000	3.3202	[13.32, 19.71]	5.5438	-10.9682
408	5	0.0006	1.2084	[3.46, 11.44]	5.5438	-1.9072
409	0	0.0000	2.0498	[7.16, 13.68]	3.0264	-7.3931
409	1	0.0000	3.5383	[17.16, 24.79]	3.0264	-17.9468
409	2	0.0000	2.8719	[13.91, 21.96]	3.0264	-14.9078
409	3	0.0000	4.0221	[23.15, 31.92]	6.0749	-21.4581
409	4	0.0000	3.8580	[23.75, 33.21]	6.0749	-22.4049
409	5	0.0000	1.4735	[5.71, 14.49]	6.0749	-4.0276
410	0	0.0756	0.5826	[-0.49, 9.38]	1.3831	-3.0636
410	1	0.0000	3.1831	[17.11, 25.79]	1.3831	-20.0704
410	2	0.0000	2.2322	[9.85, 17.81]	1.3831	-12.4460
410	3	0.0003	1.2687	[4.43, 13.49]	13.2411	4.2799
410	4	0.0000	4.4607	[27.52, 36.75]	13.2411	-18.8974
410	5	0.0000	2.1833	[11.14, 20.38]	13.2411	-2.5191
411	0	0.6002	0.1672	[-2.73, 4.66]	-11.7474	-12.7114
411	1	0.0000	1.6373	[-11.25, -4.92]	-11.7474	-3.6585
411	2	0.0000	2.0601	[-14.60, -7.68]	-11.7474	-0.6080
411	3	0.0000	3.2264	[12.93, 19.40]	0.6989	-15.4648
411	4	0.4294	0.2530	[-1.70, 3.91]	0.6989	-0.4038
411	5	0.0000	1.6370	[5.12, 11.80]	0.6989	-7.7610
412	0	0.0000	4.0959	[20.00, 27.41]	15.8943	-7.8141
412	1	0.0000	5.8315	[33.88, 42.25]	15.8943	-22.1723
412	2	0.0000	8.3585	[37.55, 43.84]	15.8943	-24.8020
412	3	0.0000	4.4039	[22.31, 29.91]	16.7053	-9.4065
412	4	0.0000	6.9798	[37.88, 45.53]	16.7053	-24.9999
412	5	0.0000	1.8915	[9.51, 19.28]	16.7053	2.3090
413	0	0.8293	0.0687	[-2.55, 3.16]	-2.8176	-3.1238
413	1	0.0020	1.0492	[-7.33, -1.78]	-2.8176	1.7353

Continued on next page

Supp. Table 3

Significant differences between single-target and sequential movements, separated by participant and sequence. Column 'd' refers to Cohen's d. Means (M sin/seq) and 95% confidence intervals of the difference are shown. NaN entries correspond to participant 402, who was excluded from this analysis because the single-target data was corrupted.

Part.	Seq.	p	d	CI	M sin	M seq
413	2	0.5520	0.1904	[-5.55, 3.03]	-2.8176	-1.5563
413	3	0.0000	2.8074	[10.64, 17.01]	5.4625	-8.3653
413	4	0.4540	0.2396	[-1.71, 3.74]	5.4625	4.4469
413	5	0.0000	3.1508	[8.92, 13.48]	5.4625	-5.7377
414	0	0.0000	3.2214	[15.28, 22.96]	5.8331	-13.2862
414	1	0.0024	1.0304	[2.88, 12.33]	5.8331	-1.7713
414	2	0.0001	1.4160	[5.41, 14.35]	5.8331	-4.0489
414	3	0.0000	1.7818	[4.67, 9.91]	6.2634	-1.0295
414	4	0.0000	3.4581	[11.79, 17.15]	6.2634	-8.2053
414	5	0.0000	1.7628	[4.79, 10.25]	6.2634	-1.2590
415	0	0.0001	1.3515	[3.76, 10.53]	-9.1225	-16.2646
415	1	0.0000	1.5878	[-11.39, -4.84]	-9.1225	-1.0090
415	2	0.0000	1.4757	[-12.40, -4.89]	-9.1225	-0.4776
415	3	0.7981	0.0815	[-2.53, 3.27]	0.9745	0.6067
415	4	0.0000	2.6142	[9.21, 15.22]	0.9745	-11.2374
415	5	0.0187	0.7858	[0.73, 7.46]	0.9745	-3.1230
416	0	0.7015	0.1225	[-5.19, 3.54]	-8.6731	-7.8499
416	1	0.0000	2.2423	[8.25, 14.89]	-8.6731	-20.2431
416	2	0.0000	2.5126	[10.99, 18.60]	-8.6731	-23.4663
416	3	0.0000	1.8555	[7.01, 14.43]	6.6132	-4.1055
416	4	0.0000	1.8597	[7.11, 14.61]	6.6132	-4.2472
416	5	0.0000	2.8508	[12.34, 19.50]	6.6132	-9.3080
417	0	0.0381	0.6800	[-6.91, -0.21]	-5.0404	-1.4814
417	1	0.0016	1.0787	[2.22, 8.70]	-5.0404	-10.4990
417	2	0.0000	2.1153	[7.57, 14.15]	-5.0404	-15.8993
417	3	0.0000	3.7001	[15.94, 22.62]	9.4835	-9.7961
417	4	0.0000	2.3970	[12.89, 22.40]	9.4835	-8.1637
417	5	0.0000	2.5852	[13.85, 23.06]	9.4835	-8.9684
418	0	0.0000	2.7058	[7.15, 11.59]	-5.3485	-14.7212
418	1	0.0121	0.8339	[-4.65, -0.61]	-5.3485	-2.7179
418	2	0.0000	1.9469	[7.08, 14.18]	-5.3485	-15.9785
418	3	0.0000	3.8272	[12.59, 17.65]	0.4238	-14.6987
418	4	0.0000	3.2585	[9.09, 13.54]	0.4238	-10.8924
418	5	0.0000	1.5352	[5.04, 12.40]	0.4238	-8.2990
419	0	0.0864	0.5583	[-8.62, 0.60]	-3.3822	0.6269
419	1	0.0000	3.5839	[22.97, 33.04]	-3.3822	-31.3894
419	2	0.0004	1.2671	[5.77, 17.86]	-3.3822	-15.1973

Continued on next page

Supp. Table 3

Significant differences between single-target and sequential movements, separated by participant and sequence. Column 'd' refers to Cohen's d. Means (M sin/seq) and 95% confidence intervals of the difference are shown. NaN entries correspond to participant 402, who was excluded from this analysis because the single-target data was corrupted.

Part.	Seq.	p	d	CI	M sin	M seq
419	3	0.1137	0.5127	[-0.55, 4.93]	5.3877	3.1980
419	4	0.0000	4.0051	[22.41, 31.10]	5.3877	-21.3691
419	5	0.0946	0.5447	[-0.49, 5.87]	5.3877	2.7005
420	0	0.0003	1.2552	[4.81, 14.87]	3.1108	-6.7274
420	1	0.0000	4.9759	[30.44, 39.43]	3.1108	-31.8252
420	2	0.0000	3.9339	[23.96, 33.28]	3.1108	-25.5064
420	3	0.0000	3.3756	[16.44, 24.20]	1.6351	-18.6833
420	4	0.0000	6.1556	[30.26, 37.32]	1.6351	-32.1559
420	5	0.0694	0.5997	[-0.41, 10.00]	1.6351	-3.1602

Supp. Table 4

Transitional coarticulation is significantly different from zero, divided by participant and sequence. Column 'd' refers to Cohen's d. Means M and 95% confidence intervals CI are shown.

Part.	Seq.	p	d	CI	M
401	0	0.0043	0.7236	[-10.46, -2.24]	-6.3495
401	1	0.0044	0.7219	[1.75, 8.19]	4.9710
401	2	0.0362	0.1204	[-8.41, 4.97]	-1.7213
401	3	0.0081	0.6614	[-15.47, -2.65]	-9.0622
401	4	0.0011	0.8598	[2.25, 7.64]	4.9481
401	5	0.7144	0.0831	[-5.92, 4.13]	-0.8918
402	0	0.0000	2.8468	[19.62, 27.34]	23.4762
402	1	0.0283	0.5309	[1.01, 15.99]	8.4963
402	2	1.0000	0.1926	[-8.49, 20.35]	5.9336
402	3	0.0161	0.5903	[1.59, 13.73]	7.6576
402	4	0.0000	1.6415	[8.25, 14.82]	11.5354
402	5	0.0000	3.5247	[12.46, 16.27]	14.3623
403	0	0.6264	0.1107	[-2.49, 4.03]	0.7712
403	1	0.0026	0.7746	[3.51, 14.22]	8.8632
403	2	0.0005	0.9285	[3.22, 9.78]	6.5021
403	3	0.0016	0.8198	[-21.08, -5.76]	-13.4163
403	4	0.0000	1.4699	[16.39, 31.71]	24.0507
403	5	0.1449	0.3400	[-1.53, 9.68]	4.0721
404	0	0.0000	2.1156	[18.48, 28.98]	23.7334
404	1	0.0000	1.7593	[18.24, 31.46]	24.8463
404	2	0.3204	0.2282	[-1.78, 5.16]	1.6912
404	3	0.0001	1.1550	[12.13, 28.65]	20.3904

Continued on next page

Supp. Table 4

Transitional coarticulation is significantly different from zero, divided by participant and sequence. Column 'd' refers to Cohen's d. Means M and 95% confidence intervals CI are shown.

Part.	Seq.	p	d	CI	M
404	4	0.0105	0.6348	[1.80, 11.89]	6.8438
404	5	0.0000	2.5462	[17.19, 24.93]	21.0562
405	0	0.0000	2.1648	[17.21, 26.70]	21.9504
405	1	0.0000	1.3399	[9.71, 20.13]	14.9215
405	2	0.8792	0.0345	[-2.11, 2.44]	0.1675
405	3	0.6727	0.0960	[-3.21, 2.12]	-0.5469
405	4	0.0000	1.7030	[8.21, 14.42]	11.3145
405	5	0.0017	0.8144	[1.91, 7.06]	4.4863
406	0	0.6999	0.0875	[-3.59, 5.25]	0.8263
406	1	0.0383	0.4977	[-13.32, -0.41]	-6.8656
406	2	0.0000	2.0558	[-19.30, -12.14]	-15.7212
406	3	0.0124	0.6174	[-11.46, -1.58]	-6.5185
406	4	0.0000	1.1790	[-12.85, -5.55]	-9.1955
406	5	0.0107	0.6331	[-12.78, -1.92]	-7.3501
407	0	0.0000	2.2862	[-21.35, -14.09]	-17.7185
407	1	0.4600	0.1686	[-6.63, 3.12]	-1.7563
407	2	0.0075	0.6689	[-9.50, -1.68]	-5.5915
407	3	0.0000	1.5058	[-13.89, -7.30]	-10.5942
407	4	0.0000	2.1288	[11.88, 18.58]	15.2277
407	5	0.0214	0.5606	[-9.23, -0.83]	-5.0285
408	0	0.0001	1.0893	[-17.28, -6.89]	-12.0855
408	1	0.5919	0.1219	[-9.06, 5.31]	-1.8724
408	2	0.0073	0.4928	[0.35, 13.44]	6.8951
408	3	0.0459	0.4778	[-8.91, -0.09]	-4.4985
408	4	0.8807	0.0340	[-2.42, 2.10]	-0.1643
408	5	0.0000	1.5974	[7.32, 13.39]	10.3579
409	0	0.0000	4.2651	[21.68, 27.03]	24.3565
409	1	0.0000	1.7681	[12.68, 21.81]	17.2477
409	2	0.2218	0.2824	[-6.03, 1.49]	-2.2678
409	3	0.0000	1.6613	[13.06, 23.30]	18.1819
409	4	0.0000	1.9456	[10.53, 17.20]	13.8687
409	5	0.0000	1.3385	[7.49, 15.55]	11.5180
410	0	0.0006	0.9210	[4.33, 13.27]	8.7969
410	1	0.4666	0.1661	[-2.51, 5.28]	1.3839
410	2	0.0003	0.9757	[3.22, 9.17]	6.1947
410	3	0.0000	1.3398	[7.72, 16.02]	11.8702
410	4	0.0119	0.6218	[-10.26, -1.45]	-5.8515
410	5	0.0000	2.5540	[16.19, 23.46]	19.8239
411	0	0.4192	0.1847	[-5.49, 2.38]	-1.5525

Continued on next page

Supp. Table 4

Transitional coarticulation is significantly different from zero, divided by participant and sequence. Column 'd' refers to Cohen's d. Means M and 95% confidence intervals CI are shown.

Part.	Seq.	p	d	CI	M
411	1	0.0000	3.9366	[30.22, 38.38]	34.3015
411	2	0.0000	1.5173	[11.36, 21.50]	16.4285
411	3	0.0006	1.1083	[8.79, 21.64]	15.2132
411	4	0.0000	7.7537	[30.64, 34.58]	32.6094
411	5	0.0002	1.0417	[5.08, 13.36]	9.2201
412	0	0.0000	1.6278	[11.10, 20.06]	15.5837
412	1	0.3134	0.2316	[-1.98, 5.84]	1.9344
412	2	0.8408	0.1736	[-3.75, 8.17]	2.2112
412	3	0.2381	0.2724	[-1.68, 6.36]	2.3384
412	4	0.0015	0.8277	[2.84, 10.22]	6.5277
412	5	0.0000	1.6231	[14.96, 27.08]	21.0213
413	0	0.0000	1.2519	[5.53, 12.14]	8.8355
413	1	0.0000	1.4438	[9.38, 18.38]	13.8771
413	2	0.6824	0.0929	[-2.25, 3.37]	0.5579
413	3	0.5148	0.1484	[-5.94, 3.08]	-1.4308
413	4	0.0000	3.0830	[18.03, 24.49]	21.2593
413	5	0.9525	0.0135	[-3.10, 2.92]	-0.0868
414	0	0.0004	0.9681	[3.59, 10.31]	6.9533
414	1	0.0973	0.1640	[-8.63, 4.15]	-2.2393
414	2	0.0002	1.0474	[3.54, 9.26]	6.4019
414	3	0.2611	0.0269	[-4.82, 4.29]	-0.2618
414	4	0.9230	0.0219	[-4.51, 4.95]	0.2213
414	5	0.0000	1.6763	[7.07, 12.55]	9.8089
415	0	0.0000	1.9205	[-17.63, -10.72]	-14.1735
415	1	0.9200	0.0228	[-3.27, 2.96]	-0.1514
415	2	0.5459	0.0368	[-6.15, 7.20]	0.5246
415	3	0.0000	1.5167	[-18.55, -9.80]	-14.1772
415	4	0.0985	0.3885	[-8.81, 0.82]	-3.9971
415	5	0.0000	1.4924	[-12.99, -6.79]	-9.8875
416	0	0.0046	0.7179	[1.86, 8.82]	5.3399
416	1	0.0007	0.9008	[3.04, 9.63]	6.3345
416	2	0.0097	0.6433	[-5.87, -0.93]	-3.3990
416	3	0.5927	0.1217	[-5.64, 3.31]	-1.1628
416	4	0.0000	1.6453	[8.28, 14.86]	11.5667
416	5	0.0020	0.7981	[-10.65, -2.78]	-6.7110
417	0	0.0000	1.5703	[8.38, 15.50]	11.9435
417	1	0.0000	2.6215	[15.62, 22.41]	19.0125
417	2	0.4685	0.1654	[-3.49, 1.67]	-0.9102
417	3	0.0074	0.6703	[1.94, 10.89]	6.4138

Continued on next page

Supp. Table 4

Transitional coarticulation is significantly different from zero, divided by participant and sequence. Column 'd' refers to Cohen's d. Means M and 95% confidence intervals CI are shown.

Part.	Seq.	p	d	CI	M
417	4	0.0000	1.2205	[8.70, 19.52]	14.1065
417	5	0.1069	0.3784	[-0.88, 8.30]	3.7124
418	0	0.0000	1.1946	[-6.36, -2.78]	-4.5732
418	1	0.2063	0.2926	[-0.88, 3.83]	1.4745
418	2	0.0000	1.5141	[5.62, 10.65]	8.1335
418	3	0.0000	1.5570	[-10.42, -5.60]	-8.0093
418	4	0.0000	1.7620	[6.93, 11.95]	9.4416
418	5	0.0002	1.2877	[8.18, 17.53]	12.8578
419	0	0.0153	0.5702	[1.08, 11.00]	6.0390
419	1	0.0003	0.9789	[-27.67, -9.77]	-18.7207
419	2	0.0037	0.5816	[-12.64, -1.37]	-7.0058
419	3	0.0083	0.6587	[-13.52, -2.29]	-7.9057
419	4	0.0000	1.4655	[-11.20, -5.78]	-8.4912
419	5	0.0021	0.7940	[2.60, 10.06]	6.3282
420	0	0.0000	1.9958	[17.38, 28.02]	22.6998
420	1	0.0137	0.6077	[1.13, 8.72]	4.9287
420	2	0.0116	0.6248	[1.49, 10.38]	5.9349
420	3	0.0000	0.9944	[27.95, 77.67]	52.8117
420	4	0.1201	0.3639	[-0.83, 6.62]	2.8945
420	5	0.0000	1.3097	[13.35, 28.19]	20.7665

References

[1] V S Afraimovich, V P Zhigulin, and M I Rabinovich. "On the Origin of Reproducible Sequential Activity in Neural Circuits". In: *Chaos (Woodbury, N.Y.)* 14.4 (Dec. 2004), pp. 1123–1129. ISSN: 1054-1500. DOI: 10.1063/1.1819625.

[2] Valentin Afraimovich, Dario Cuevas, and Todd Young. "Sequential Dynamics of Master–Slave Systems". In: *Dynamical Systems* 28.2 (June 2013), pp. 154–172. ISSN: 1468-9367, 1468-9375. DOI: 10.1080/14689367.2013.777398. (Visited on 09/28/2021).

[3] Peter beim Graben et al. "Metastable Resting State Brain Dynamics". In: *Frontiers in Computational Neuroscience* 13 (Sept. 2019). ISSN: 1662-5188. DOI: 10.3389/fncom.2019.00062. (Visited on 02/24/2025).

[4] Tilen Brecelj and Tadej Petrič. "Stable Heteroclinic Channel Networks for Physical Human–Humanoid Robot Collaboration". In: *Sensors* 23.3 (Jan. 2023), p. 1396. ISSN: 1424-8220. DOI: 10.3390/s23031396. (Visited on 02/24/2025).

[5] Dario Cuevas Rivera, Sebastian Bitzer, and Stefan J. Kiebel. "Modelling Odor Decoding in the Antennal Lobe by Combining Sequential Firing Rate Models with Bayesian Inference". In: *PLoS Comput Biol* 11.10 (Oct. 2015), e1004528. DOI: 10.1371/journal.pcbi.1004528. (Visited on 11/11/2015).

[6] Stefan J. Kiebel et al. “Recognizing Sequences of Sequences”. In: *PLoS Comput Biol* 5.8 (Aug. 2009), e1000464. DOI: 10.1371/journal.pcbi.1000464. (Visited on 08/05/2014).

[7] M. Rabinovich et al. “Dynamical Encoding by Networks of Competing Neuron Groups: Winnerless Competition”. In: *Physical Review Letters* 87.6 (July 2001), p. 068102. DOI: 10.1103/PhysRevLett.87.068102. (Visited on 02/04/2015).

[8] Mikhail I. Rabinovich et al. “Chunking Dynamics: Heteroclinics in Mind”. In: *Frontiers in Computational Neuroscience* 8 (Mar. 2014). ISSN: 1662-5188. DOI: 10.3389/fncom.2014.00022. (Visited on 11/26/2024).

[9] Natasha Rouse and Kathryn Daltorio. “Stable Heteroclinic Channel-Based Movement Primitives: Tuning Trajectories Using Saddle Parameters”. In: *Applied Sciences* 14.6 (Jan. 2024), p. 2523. ISSN: 2076-3417. DOI: 10.3390/app14062523. (Visited on 11/26/2024).

[10] Emanuel Todorov. “Stochastic Optimal Control and Estimation Methods Adapted to the Noise Characteristics of the Sensorimotor System”. In: *Neural Computation* 17.5 (May 2005), pp. 1084–1108. ISSN: 0899-7667. DOI: 10.1162/0899766053491887. (Visited on 06/08/2022).
